# Nucleotide salvage, genome instability, and potential therapeutic applications

**DOI:** 10.1093/nar/gkag099

**Published:** 2026-02-10

**Authors:** Pengcheng Wang, Chen Wang, Yinsheng Wang

**Affiliations:** Department of Chemistry, University of California Riverside, Riverside, CA 92521-0403, United States; Department of Chemistry, University of California Riverside, Riverside, CA 92521-0403, United States; Department of Chemistry, University of California Riverside, Riverside, CA 92521-0403, United States

## Abstract

Nucleotide salvage is crucial for maintaining DNA replication when *de novo* nucleotide synthesis is limited, but this metabolic flexibility poses potential threats to genome stability. Salvage kinases phosphorylate nucleosides broadly, allowing for oxidized and alkylated 2′-deoxynucleosides as well as posttranscriptionally modified ribonucleosides to enter the 2′-deoxynucleoside triphosphate (dNTP) pool. The ensuing contamination of the dNTP pool and the subsequent incorporation of modified nucleotides into genomic DNA promote mutagenesis, induce replication stress, elicit double-strand breaks, and disrupt epigenetic signaling. Although only a small subset of modified nucleosides have been assessed for salvage and genomic incorporation, the scope of salvageable substrates is probably much wider, with significant implications in mutational burden, chromatin instability, and epigenetic regulation. This overlooked aspect of genome instability is especially relevant in biological contexts of high salvage activity or elevated nucleoside damage, including chronic inflammation, cancer, aging, and dietary/microbiome exposures. Emerging evidence links salvage metabolism to tumor progression, where incorporation of salvage-derived nucleotides may contribute to unexplainable mutational signatures detected in cancers, such as gastrointestinal cancer. Recognizing salvage as a hidden source of mutagenesis reshapes our understanding of genome instability and provides potential opportunities for disease prevention, diagnosis, and therapeutic intervention.

## Graphical Abstract

**Graphical Abstract:**
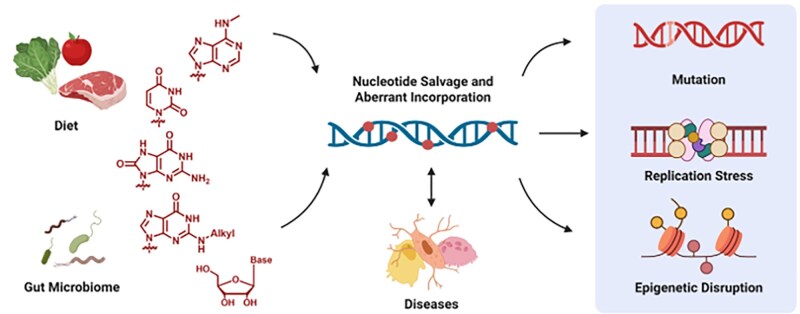

## Introduction

Accurate DNA replication and repair require a balanced supply of canonical 2′-deoxyribonucleoside triphosphates (dNTPs). Human cells maintain dNTP pool through the coordinated action of two metabolic routes, i.e. *de novo* nucleotide biosynthesis, which converts simple metabolites into nucleotides, and the nucleotide salvage pathway, which recycles preformed nucleobases and nucleosides originating from endogenous turnover, extracellular nutrients, or the gut microbiome [1, 2].

*De novo* nucleotide biosynthesis is metabolically expensive, consuming substantial amounts of ATP, and is tightly regulated through multiple mechanisms, such as transcriptional control, posttranscriptional modifications, feedback inhibition, and the assembly of multienzyme complexes [2]. In contrast, the salvage pathway produces nucleotides in an energy-efficient manner and is generally considered beneficial by reducing waste and facilitating genome preservation under replication stress and/or resource scarcity [2]. Additionally, nucleotide salvage was shown to be important for telomere length maintenance [3, 4]. Although neither *de novo* synthesis nor salvage pathway possesses intrinsic quality control mechanisms, *de novo* synthesis is tightly regulated to maintain dNTP abundance and pool balance, whereas salvage lacks upstream regulatory checkpoints and directly activates preformed nucleosides with a broad substrate scope [1, 2].

**Figure 1.**
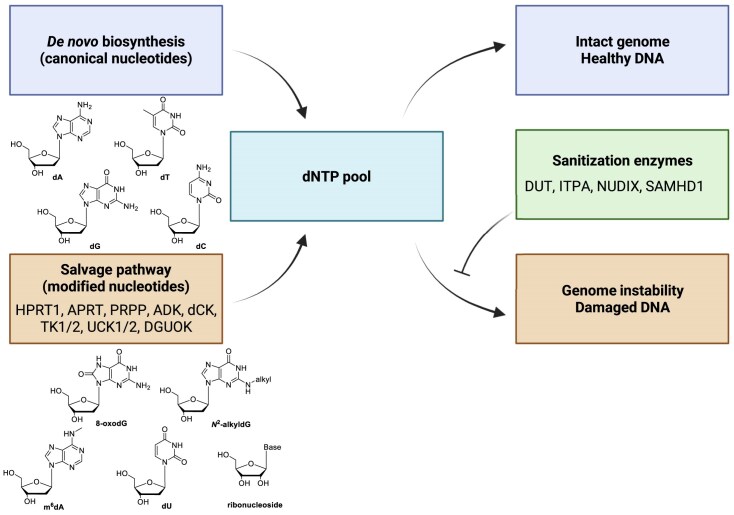
Salvage-driven entry of modified nucleosides into the genome. *De novo* synthesis generates canonical nucleotides, whereas salvage pathways recycle modified nucleosides directly into the dNTP pool. The chemical structures illustrate the canonical (top) and modified (bottom) nucleosides. Sanitization enzymes safeguard the integrity of the dNTP pool.

A defining feature of salvage is its high substrate tolerance. Human nucleoside kinases phosphorylate purine and pyrimidine nucleosides with limited discrimination against chemical modifications [1, 2]. As a result, noncanonical or damaged nucleotides can be metabolically activated to yield the corresponding dNTPs, thereby resulting in their erroneous incorporation into genomic DNA (Fig. 1) [5–17]. These include oxidized nucleosides [e.g. 8-oxo-7,8-dihydro-2′-deoxyguanosine (8-oxodG)] [6–8], alkylated nucleosides (e.g. *N*^2^-alkyl-dG) [5, 8, 14–17], deaminated nucleosides [e.g. 2′-deoxyuridine (dU)] [9], a nucleoside derived from a posttranscriptionally modified ribonucleoside via the salvage pathway [i.e. *N*^6^-methyl-2′-deoxyadenosine (*N*^6^-MedA), through salvage-mediated conversion of *N*^6^-methyladenosine (m^6^A) into *N*^6^-MedATP and its subsequent incorporation into DNA] [10, 11, 18, 19], and ribonucleotides when the balance between ribonucleoside triphosphate (rNTP) and dNTP pools is disturbed [12, 13, 20]. Upon incorporation, these aberrant nucleotides may evade DNA repair, induce DNA strand breaks, alter chromatin structure, and promote mutagenesis.

Recent studies suggest that salvage-mediated entry of modified nucleotides into genomic DNA constitutes an overlooked source of genome instability. This perspective synthesizes recent findings linking nucleotide salvage to genome instability and explores its consequences on mutagenesis, replicative stress, and epigenetic regulation. By reevaluating this metabolic recycling process, we reveal a crucial link between metabolism and genomic integrity, with implications in inflammation, cancer, and natural process of aging.

## Nucleotide salvage and the dNTP pool

Salvage pathways maintain nucleotide homeostasis. In human cells, salvage encompasses enzymes located in both the cytosol and mitochondria that convert nucleobases or nucleosides into nucleoside monophosphates (NMPs), which are subsequently phosphorylated to nucleoside diphosphates and NTPs [2, 21, 22]. Unlike *de novo* biosynthesis, salvage is primarily constrained by substrate availability and kinase expression, rendering it a metabolically efficient, albeit potentially less discriminating source of dNTPs.

**Figure 2.**
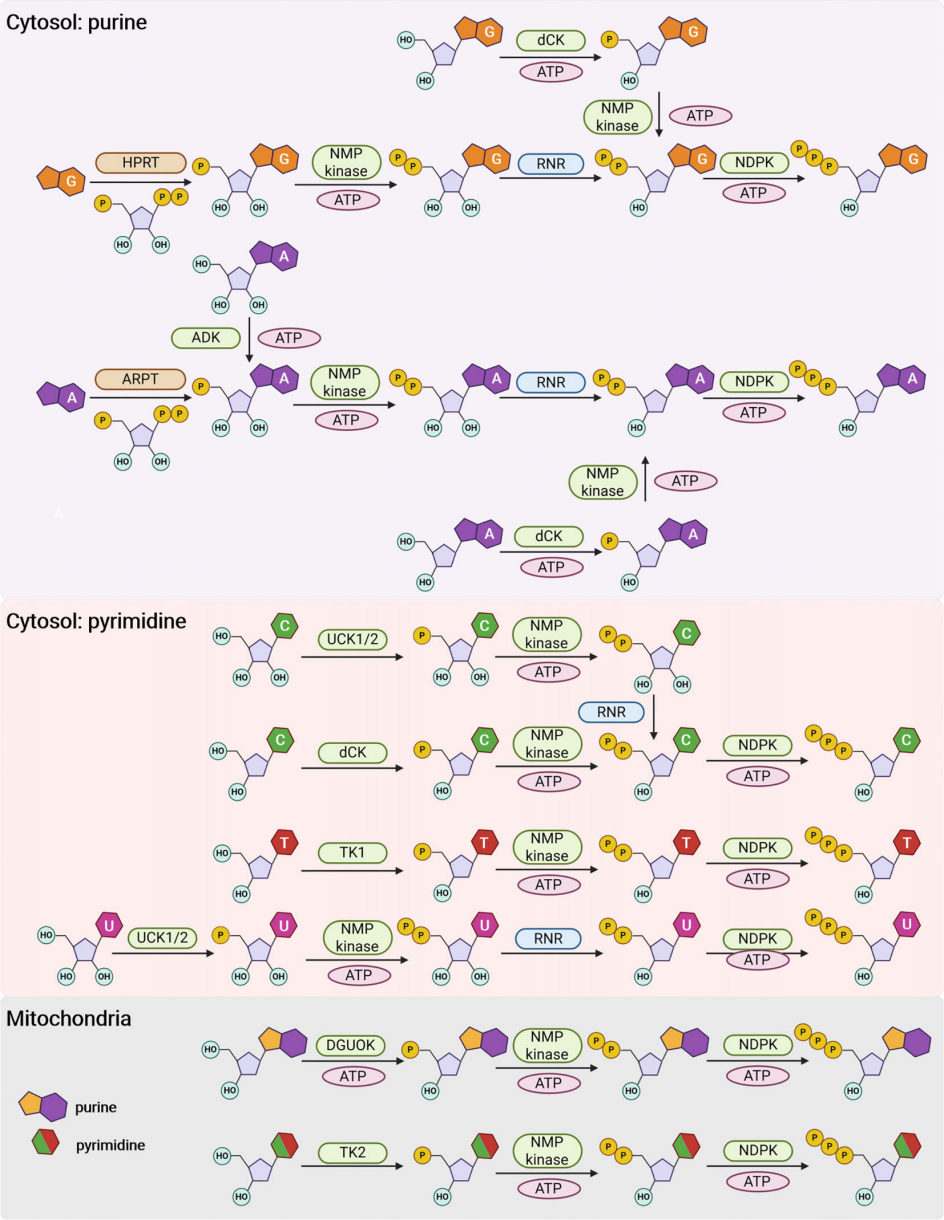
Salvage pathways of purines and pyrimidines to dNTPs in human cells. In the cytosol, purine bases are salvaged via PRPP-dependent phosphoribosyltransferases, with HPRT1 generating IMP and GMP, and APRT producing AMP. In parallel, purine and pyrimidine nucleosides are phosphorylated by ADK, dCK, TK1, and UCK1/2. Mitochondrial nucleotide salvage is mediated primarily by deoxyguanosine kinase (DGUOK) and TK2. Salvage-derived NMPs are sequentially converted to diphosphates and triphosphates by NMP kinases and nucleoside diphosphate kinase (NDPK), with ribonucleoside diphosphates reduced to their 2′-deoxy counterparts by class I ribonucleotide reductase (RNR).

Human nucleotide salvage recycles purine and pyrimidine bases and nucleosides derived from endogenous nucleic acid turnover or extracellular sources to sustain nucleotide pools (Fig. 2). Purine bases are salvaged through phosphoribosyltransferase-dependent reactions, with hypoxanthine-guanine phosphoribosyltransferase (HPRT1) catalyzing the conversions of hypoxanthine and guanine to inosine monophosphate (IMP) and guanosine monophosphate (GMP), respectively, and adenine phosphoribosyltransferase (APRT) converting adenine to adenosine monophosphate (AMP), where phosphoribosyl pyrophosphate (PRPP) serves as the ribose donor [23–25]. In parallel, purine and pyrimidine nucleosides are recycled by nucleoside kinases, including adenosine kinase (ADK), deoxycytidine kinase (dCK), thymidine kinases 1 and 2 (TK1/TK2), and uridine–cytidine kinases 1 and 2 (UCK1/2), generating NMPs that support ribonucleotide and deoxyribonucleotide syntheses [26–30]. Notably, uracil phosphoribosyltransferase—which catalyzes uracil salvage in plants, protozoa, and bacteria—lacks detectable catalytic activity in humans [31]; physiological pyrimidine salvage in human cells, therefore, proceeds exclusively through nucleoside-based pathways. While unmodified uracil cannot enter the nucleotide pool via the salvage pathway, anti-cancer drug 5-fluorouracil could be activated by orotate phosphoribosyltransferase to yield 5-FUMP, which can be converted into 5-FdUTP and incorporated into DNA [32].

Mitochondrial nucleotide salvage is mediated primarily by DGUOK and TK2, sustaining mitochondrial DNA replication and repair [22, 33–35]; mutations in genes encoding these two proteins could give rise to mitochondrial DNA depletion syndrome and neurogastrointestinal encephalomyopathy [33–35].

Ribonucleoside diphosphates (rNDPs) produced through salvage are converted to their 2′-deoxy counterparts by class I ribonucleotide reductase (RNR), which reduces rNDPs to dNDPs [36]. These dNDPs are subsequently phosphorylated by NDPK to yield the corresponding dNTPs [37].

The activities of these enzymes are dynamically regulated at both the transcriptional and posttranslational levels, and nucleotide salvage is often elevated under disease or stress conditions. For instance, the MYC oncoprotein broadly upregulates nucleotide metabolic enzymes, including those involved in the salvage pathway [38], while hypoxia-inducible factors (e.g. HIF-1α) reprogram purine and pyrimidine metabolisms in hypoxic tumor microenvironments [39, 40]. As such, salvage activity is generally elevated in cancerous and inflamed tissues, increasing the likelihood that noncanonical substrates enter the dNTP pool.

Because neither *de novo* nor salvage pathways proofread nucleotide chemical integrity, genome protection after metabolic activation depends largely on nucleotide sanitization enzymes, which can be divided into four super families, all-β dUTPases, ITPases, all-α NTP pyrophosphatases, and NUDIX hydrolases [41]. These enzymes degrade inappropriate nucleotides before their utilization by DNA polymerases, thereby limiting mutagenic or replication-perturbing events. In human cells, key representatives of these families include DUT (dUTPase) [42], ITPA [43], the NUDIX hydrolases NUDT1 (MTH1) [44, 45], NUDT15 [46], and NUDT16 [47], and the all-α dNTP triphosphohydrolase SAMHD1 [48], which collectively safeguard dNTP pool integrity (Fig. 1). DUT hydrolyzes dUTP to prevent uracil incorporation [42], while ITPA removes deaminated purine triphosphates, e.g. 2′-deoxyinosine triphosphate (dITP), that would otherwise promote mispairing and replication stress [43]. Oxidized purine triphosphates, including 8-oxo-dGTP and 2-hydroxy-dATP, are efficiently hydrolyzed by NUDT1 (MTH1) [44, 45], whereas SAMHD1 regulates overall dNTP abundance and pool balance [48], and is involved in the sanitization of therapeutic nucleotide analogs [49–51]. Additional NUDIX enzymes, e.g. NUDT15, sanitize thiopurine metabolites and are particularly relevant in the context of thiopurine therapy [46]. Collectively, these enzymes form a defense system that intercepts aberrant nucleotides to safeguard genome integrity. However, when nucleotide salvage is upregulated and/or sanitization enzymes are overwhelmed or dysregulated, such as in disease contexts [52–54], this defense becomes insufficient, resulting in elevated incorporation of aberrant nucleotides into genomic DNA.

Perturbations in dNTP pool composition and balance provide a direct mechanistic link between salvage activity and genome instability. Imbalanced nucleotide pools lead to augmented base misincorporation rates and diminished polymerase fidelity [55], whereas dNTP pool depletion can impede DNA polymerases and induce replication fork stalling [56]. Under these conditions, increased reliance on translesion synthesis (TLS) polymerases, including Pol κ and Pol η, promotes the incorporation of noncanonical substrates, further destabilizing genomic integrity [56]. These effects establish a metabolism–DNA replication paradigm where aberrant salvage activity confers mutagenesis, replication stress, and genome instability.

## Incorporation of aberrant nucleotides

### Oxidized nucleosides

Oxidative stress has long been found to induce DNA oxidation, resulting in a plethora of DNA modifications [57]. Among the oxidatively generated DNA adducts, 8-oxodG has received the most attention due to the lowest oxidation potential of dG among the four canonical 2′-deoxynucleosides [57]. Hence, the occurrence and biological consequences of 8-oxodG salvage have been extensively investigated [6, 54, 58–63]. Research indicates that 8-oxodG can be salvaged into the nucleotide pool, where it is hydrolyzed by purine nucleoside phosphorylase (PNP) to yield 8-oxoguanine instead of being directly phosphorylated. This process then leads to the formation of the corresponding nucleoside triphosphates, 8-oxorGTP and 8-oxodGTP [6]. The 8-oxodGTP can be utilized by DNA polymerases and incorporated into genomic DNA [58, 60–63]. For instance, DNA polymerase β (Pol β) can incorporate 8-oxodGTP with the formation of 8-oxodG:dC and 8-oxodG:dA mispairs within DNA trinucleotide repeat [59]. This also holds true for Pol η and Pol κ, which can efficiently insert 8-oxodGMP opposite a template dA [60, 63, 64]. The reported level of salvaged 8-oxodG incorporation is of the same order of magnitude as basal level of genomic 8-oxodG [6, 65]. The measurement, however, was conducted under experimental conditions involving near-saturating extracellular 8-oxodG, and thus may represent an upper bound for salvage-mediated incorporation [6].

Under normal conditions, this threat is mitigated by NUDT1 (MTH1), which hydrolyzes 8-oxodGTP to 8-oxodGMP [44, 45]. When nucleotide sanitization is inadequate, e.g. during oxidative stress or elevated salvage flux, misincorporation increases substantially, promoting mutagenesis.

### Alkylated lesions

Alkylated DNA lesions represent another major type of extensively studied DNA damage [57, 66]. The ubiquitous presence of alkylating agents in the environment and inside cells renders this type of lesions generally unavoidable [67, 68]. Aside from being generated by alkylating agents, DNA alkylation may arise from the salvage of alkylated nucleosides and their subsequent incorporation into genomic DNA. Indeed, Spratt and coworkers [5] demonstrated that *N*^2^-substituted dGTP derivatives with methyl (Me), *n*-butyl (*n*Bu), benzyl or 4-ethynylbenzyl group are robust substrates for human DNA Pol κ, as evidenced by both *in vitro* and cellular experiments. In addition, work from our laboratory showed that feeding cultured human cells with *N*^2^-MedG and *N*^2^-*n*BudG resulted in augmented levels of the two modified nucleosides in genomic DNA [8, 14]. Interestingly, Spratt and we found that incorporation of *N*²-alkyl-dG occurs preferentially during early S-phase DNA replication [15, 69]. In addition, DNA Pol κ and, to a lesser extent, Pol η facilitate the incorporation of *N*^2^-MedG and *N*^2^-*n*BudG into genomic DNA [5, 8, 14]. Furthermore, depletion of ALKBH3 in cultured human cells led to increased accumulation of *N*^2^-MedG and *N*^2^-*n*BudG [8].

### Food consumption

Cooking food, especially at high temperatures, has long been shown to be associated with adverse health consequences, including higher tendency to develop colorectal cancer and diabetes [70]. Conventionally, DNA adducts induced by chemical species generated during cooking process, e.g. polycyclic aromatic hydrocarbons and heterocyclic aromatic amines, are of primary concern when considering genotoxic agents induced by cooking [71]. The DNA in food itself, however, received little attention. High-temperature treatment can produce oxidized purines, thymine glycol, and hydrolytic deamination products [72, 73]. Consequently, dietary DNA can carry pre-existing lesions formed during cooking and/or food processing/storage. Upon digestion, these pre-existing modified nucleosides can be absorbed, enter salvage pathways, and become available for incorporation into DNA. A recent study by the Kool laboratory showed that HeLa cells exhibit elevated levels of DNA double-strand breaks (DSBs) upon incubation with damaged nucleosides, e.g. dU, 5-hydroxy-2′-deoxyuridine (5-OHdU), 5-hydroxy-2′-deoxycytidine (5-OHdC), 5,6-dihydro-thymidine (DH-dT), and thymidine glycol (dTg) [9]. This finding supports that deaminated and oxidatively damaged nucleosides can be taken up into cells, metabolically activated and incorporated into genomic DNA, ultimately resulting in DSBs [9]. However, it is important to note that dTg nucleotide is more difficult to be utilized by polymerases during DNA synthesis [74]. Furthermore, oral administration of mice with dU led to augmented levels of dU in genomic DNA isolated from small intestine [9]. Therefore, salvage of damaged DNA components from food can be a potential contributor to genome instability [9].

### Ribonucleotide oversupply

rNTPs are normally present at levels that are orders of magnitude higher than dNTPs [75]. Although DNA polymerases are capable of excluding rNTPs, such exclusion is incomplete; hence, even high-fidelity replicative DNA polymerases can incorporate rNMPs during DNA synthesis [12, 13]. For instance, human lagging-strand replicative polymerase Pol δ was shown to incorporate approximately one rNTP in every 2 000 dNTPs, although the experiment was performed *in vitro* using purified protein [12]. Moreover, a substantial amount of inserted rNTPs by leading-strand replicative polymerase Pol ε escape 3′→5′ exonucleolytic proofreading [13]. In addition to replicative polymerases, human Pol η is capable of incorporating rCMP opposite dG and 8-oxodG [76]. The misincorporation of ribonucleotides into genomic DNA has been extensively investigated, and its prevalence and biological implications have been reviewed elsewhere [77, 78].

### RNA-derived modifications

Different from its DNA counterpart, RNA carries a diverse array of chemical modifications, with more than 150 distinct modifications identified to date [79]. In addition, some of these modifications occur at high stoichiometries [80, 81]. Degradation of RNA continuously releases these modified nucleosides, which can be recycled through salvage pathways to produce not only rNTPs, but also chemically modified dNTPs. Once in the nucleotide pool, these aberrant substrates may be utilized by DNA polymerases and incorporated into genomic DNA.

This is the case for *N*^6^-MedA. Several reports in 2015 proposed that *N*^6^-MedA can act as an epigenetic mark in eukaryotes [82–84], and aberrant deposition of *N*^6^-MedA was shown to modulate gene expression and contribute to the proliferation of glioblastoma cells [19, 85, 86]. This role of *N*^6^-MedA, however, remains controversial, where multiple studies revealed extremely low levels of *N*^6^-MedA in mammalian genomes [19, 87, 88]. Moreover, recent studies indicate that m^6^A from RNA degradation can be metabolically activated to yield *N*^6^-MedATP, which can be incorporated into genomic DNA by DNA polymerases [10, 11]. Importantly, isotope tracing experiments revealed that a substantial portion of the genomic *N*^6^-MedA in cultured mammalian cells originates not from DNA methyltransferase-mediated methylation, but rather from m^6^A [10, 11]. These findings challenge the view of *N*^6^-MedA as an epigenetic mark in mammalian cells, and suggest that *N*^6^-MedA in DNA may arise from a byproduct of metabolic recycling.

Consistent with stringent cellular control over this process, a recent study by Chen *et al.* [18] identified an active checkpoint that limits aberrant DNA *N*^6^-MedA arising from RNA m^6^A turnover. In particular, the adenosine deaminase-like protein ADAL catabolizes *N*^6^-MedAMP *in vivo*, thereby preventing its accumulation and subsequent incorporation into DNA. Loss of ADAL results in elevated levels of free m^6^A species and augmented misincorporation of *N*^6^-MedAMP into DNA, which is suppressed by AK1 depletion [18]. Together, these findings support a model where RNA-derived m^6^A can enter salvage pathways, but is normally intercepted by dedicated sanitization mechanisms, thereby minimizing the incorporation of *N*^6^-MedA into genomic DNA. In addition, the salvage pathway may constitute an important route for incorporating other RNA-derived modifications into DNA.

## Biological consequences of salvage-driven incorporation

**Figure 3.**
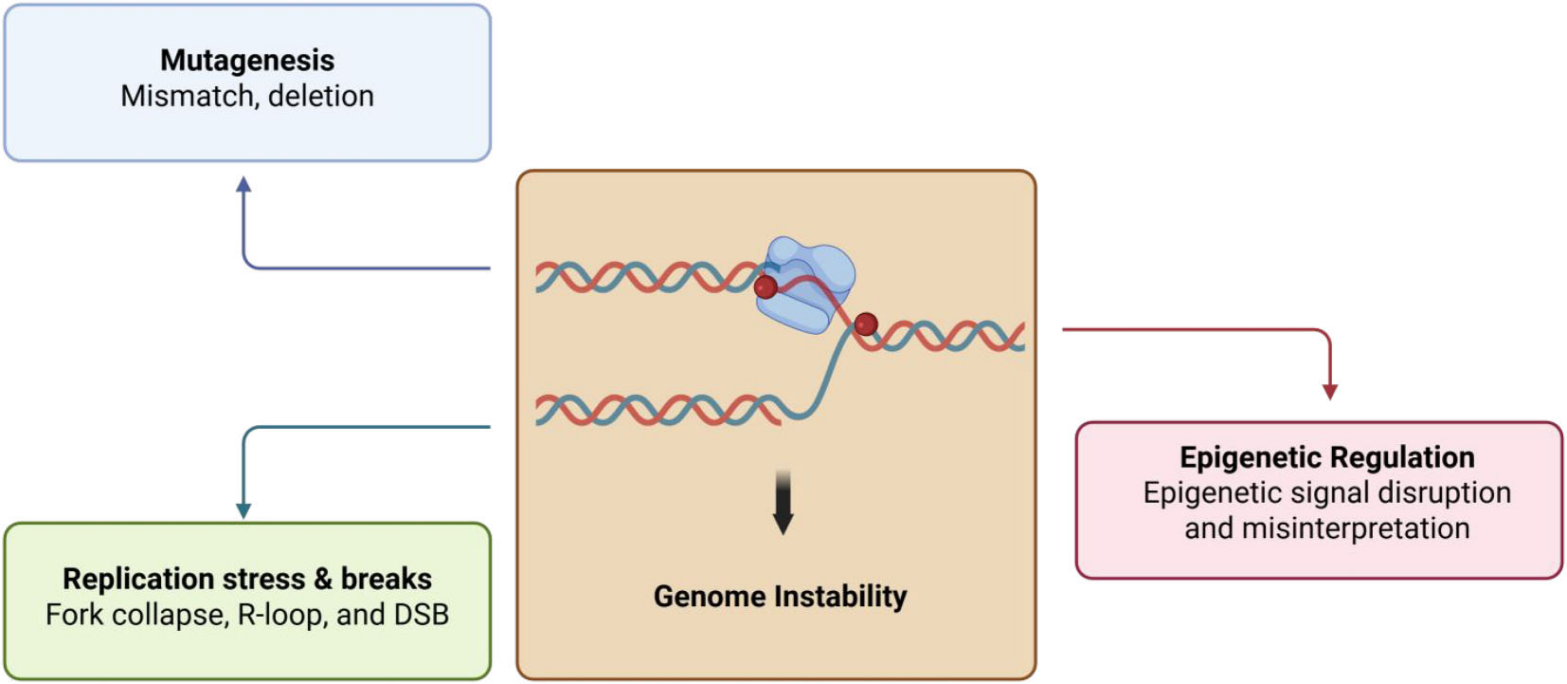
Biological consequences of salvage-driven incorporation.

The incorporation of salvage-derived modified nucleotides into genomic DNA may confer adverse biological consequences, including induction of mutagenesis, replication stress and strand breaks, as well as disruption of epigenetic regulation, all of which can ultimately lead to genome instability (Fig. 3).

### Mutagenesis

Once modified nucleotides are incorporated into DNA, the DNA replication machinery often struggles to maintain fidelity. To prevent replication fork collapse, cells rely on TLS DNA polymerases, e.g. Pol η, Pol ι, Pol κ, Pol ζ, and REV1 [56, 89]. These polymerases can accommodate bulky or noncanonical nucleobases into their active sites, facilitating bypass of those lesions that stall replicative polymerases, albeit sometimes at the expense of fidelity. For example, Pol κ often inserts mismatched nucleotides opposite oxidized purines [90], while REV1 specializes in incorporating dCMP regardless of the template base [91]. In addition, modified bases can pair with incorrect nucleotides. A well-studied example is 8-oxodG, which can adopt the *syn N*-glycosidic bond configuration, facilitating Hoogsteen pairing with dA, though it pairs correctly with dC when in the *anti* configuration [92]. As such, 8-oxodG can result in G → T transversion [93].

It should be noted that TLS polymerases are required for the error-free bypass of *N*^2^-alkyldG lesions [94–97]. In this vein, our laboratory showed that loss of Pol ι, Pol κ, or REV1 diminishes bypass efficiency and results in substantial frequencies of G → A transition and G → T transversion mutations at the site of *N*^2^-alkyldG lesions in mammalian cells [95, 97]. Moreover, simultaneous depletion of Pol ζ together with Pol κ or Pol ι further aggravates the frequencies of these mutations, suggesting the cooperation of TLS polymerases in mitigating replication errors [95].

Apart from nucleobase modifications, unrepaired ribonucleotides embedded in DNA can promote genome instability. In mammalian cells, ribonucleotides incorporated into genomic DNA are primarily removed by RNase H2-dependent ribonucleotide excision repair, where impairment in RNase H2 activity leads to ribonucleotide accumulation and increased DNA damage [98, 99]. In biochemical and cellular systems, human topoisomerase I (Top1) exhibits endoribonuclease activity and cleaves ribonucleotide-containing DNA, generating strand breaks and repair intermediates [100]. Moreover, defective ribonucleotide excision repair in mammals is associated with the ID4 signature mutation—an insertion-deletion mutation detected in human cancers characterized by short 2–5 base pair deletion—occurring at a TNT sequence motif, suggesting a mechanism involving TOP1 activity at ribonucleotide sites embedded in the genome [101].

### Replication stress and DNA strand breaks

Lesions that escape from repair can stall DNA polymerases and block replication fork progression. Persistent stalling promotes fork reversal, collapse and ultimately DSB induction. Oxidatively generated DNA lesions such as 8-oxodG were shown to elicit structural changes around the replication fork, interfering with DNA replication [102, 103]. *N*^2^-alkyldG lesions also impede DNA replication with lower bypass efficiencies [95] and were recently linked to R-loop accumulation [16], leading to genome instability. In addition, salvage of damaged nucleosides, e.g. dU, 8-oxodG, 5-OHdU and 5-OHdC, could give rise to high activities of base excision repair (BER), resulting in DSBs [9]. Feeding cells with dU, 5-OHdU and 5-OHdC also leads to chromosomal aberrations, although this effect was not significant for 8-oxodG [9]. Moreover, mice administered with dU exhibited elevated DNA repair activity and increased level of DSBs [9]. Apart from BER, additional repair pathways are also involved in mitigating salvage-elicited genomic instability.

Mismatch repair (MMR) corrects mispairs like 8-oxodG:dA [102], preventing mutagenic G→ T transversions, where oxidized dNTP pool was shown to result in genomic instability in MMR-deficient *Msh2*^−/−^ mouse embryonic fibroblasts [104]. In addition, nucleotide excision repair (NER) excises bulky and minor-groove lesions like *N*^2^-alkyl-dG [105], which can otherwise be bypassed accurately by Pol κ [94–97]. Under conditions of high salvage flux, however, the capacity of MMR and NER to process these lesions may be overwhelmed, exacerbating replication stress and genome instability.

Ribonucleotides embedded in DNA confer structural distortions that hinder replication and transcription. Even a single ribonucleotide alters DNA electrostatic potential, groove width, and duplex elasticity, and RNA’s intrinsically high hydrolytic instability accelerates single-strand break formation [98, 106].

### Epigenetic regulation

An additional layer of complexity arises when RNA-derived modifications, such as m^6^A, enter the nucleotide pool via salvage. Once converted into *N*^6^-MedATP and incorporated into DNA [10, 11], this aberrant nucleotide may confer epigenetic-like effects. Indeed, *N*^6^-MedA has been reported as an emerging DNA modification capable of modulating gene expression, chromatin organization, and processes such as DNA replication and recombination in mammals [19, 87, 107]. Notably, functional roles for DNA *N*^6^-MedA have also been described in certain pathophysiological settings, including glioblastoma [19, 85]. In this vein, salvage-mediated misincorporation of *N*^6^-MedA may perturb normal epigenetic regulation by introducing noncanonical modifications into DNA, rather than representing a *bona fide* epigenetic mark.

Likewise, some oxidatively damaged nucleosides, e.g. 8-oxodG, are also implicated in modulating G-quadruplex folding in gene promoters to regulate gene expression [108–110], and were found to crosstalk with histone epigenetic marks and DNA methylation [111–114]. In this context, 8-oxodG at CpG site was shown to alter DNA methyltransferase binding and modulate DNA cytosine methylation *in vitro* [112, 114]. In addition, 8-oxodG arising from histone demethylation-induced ROS was shown to recruit 8-oxoguanine-DNA glycosylase 1 and topoisomerase 2β, which elicit chromatin and DNA conformational changes that are essential for estrogen-induced transcription [113]. As such, salvage-driven recycling of 8-oxodG into the nucleotide pool, followed by misincorporation into DNA, may not only compromise genome integrity, but also disrupt epigenetic signaling.

## Relevant biological contexts

**Figure 4.**
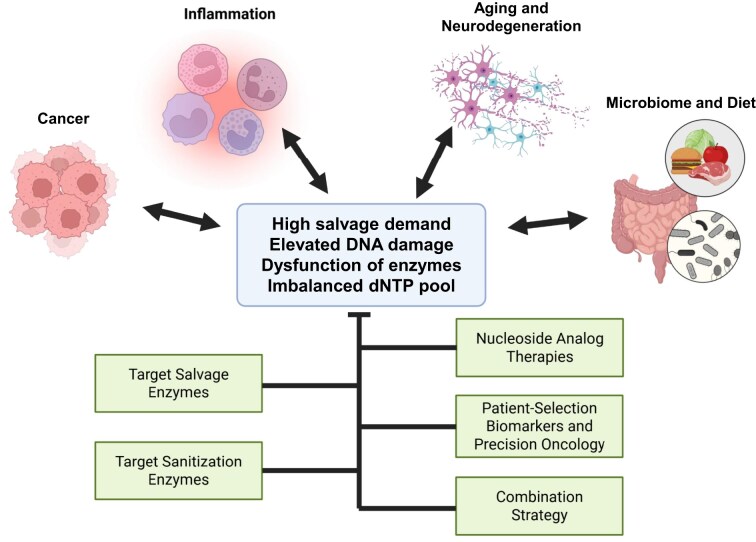
Nucleotide salvage in relevant biological contexts and potential therapeutic implications.

The detrimental impact of the salvage-driven incorporation of aberrant nucleotides is not uniform, which depends on cell type, metabolic state, environmental exposure and the capacity of DNA repair and nucleotide sanitization systems. The salvage-driven genome instability may be more pronounced in biological contexts of high salvage demand or elevated nucleoside damage, such as in rapidly proliferating cancer cells and aged tissues, during chronic inflammation, and through exposure from diet and/or gut microbiome (Fig. 4).

### Cancer

Cancer cells frequently upregulate nucleotide salvage to meet elevated dNTP demand associated with rapid proliferation while simultaneously experiencing oxidative stress, hypoxia, inflammation, high nucleic acid turnover, and altered nucleoside transporter activity [115]. Oncogene amplification (e.g. *MYC*) and activation (e.g. *HIF1A*) induce expression of salvage enzymes (TK1, dCK, HPRT1, and UCK2) [40, 116, 117], thereby increasing intracellular access to preformed modified nucleosides.

Elevated salvage flux increases the probability that chemically modified nucleosides are phosphorylated to triphosphates and incorporated into DNA. In tumors, this risk is exacerbated by chronic ROS production [118], dysregulated nucleotide sanitization due to altered NUDT1 or SAMHD1 activity [49, 119], and increased reliance on TLS polymerases [120, 121]. Together, these features create an environment that is conducive for salvage-driven mutagenesis and genome instability.

### Chronic inflammation

Chronically inflamed tissues generate high levels of reactive oxygen and nitrogen species, as well as hypohalous acids, which damage endogenous DNA, RNA, and dietary or microbial nucleic acids [122–125]. Salvage pathways can convert these modified nucleosides into dNTPs, increasing the likelihood of their incorporation into genomic DNA. Recurrent cycles of damage and repair in conditions such as inflammatory bowel disease, gastritis, and chronic hepatitis may therefore create a cellular environment particularly susceptible to salvage-driven genome instability.

### Aging and neurodegeneration

Aging is associated with diminished expression and/or activity of nucleotide sanitization enzymes, e.g. NUDT1 [53], mitochondrial dysfunction [21, 126], increased ROS burden [127], and imbalanced dNTP pool arising from a proof-reading-deficient mutant of replicative DNA polymerase in mitochondria (i.e. Pol γ^D257A^) [128]. This mutant Pol γ-elicited dNTP pool imbalance, however, could not reproduced in a subsequent study [129]. These age-related changes collectively confer heightened vulnerability to salvage-derived lesions. Post-mitotic tissues such as neurons, which rely heavily on salvage pathways due to limited *de novo* nucleotide synthesis [130, 131], may be especially susceptible to incorporation of noncanonical nucleotides [132]. Although neurons do not divide, mitochondrial biogenesis and robust DNA repair required to preserve cell integrity can create opportunities for modified nucleotides to be incorporated into mitochondrial and nuclear DNA [132–134]. Elevated levels of oxidized nucleosides observed in neurodegenerative disorders such as Parkinson’s disease and Alzheimer’s disease raise the possibility that salvage-mediated incorporation of modified nucleotides contributes to mitochondrial genome instability and impaired neuronal survival [135–137].

### Microbiome- and diet-derived nucleosides

The gut microbiome represents a continuous source of chemically modified nucleosides, including deaminated purines, alkylated bases, and oxidized products arising from microbial metabolism and host–microbe redox interactions [138, 139]. These nucleosides can traverse the intestinal epithelium and enter systemic circulation [9], rendering them accessible to salvage pathways in mammalian systems.

Dietary intake constitutes an additional exogenous source of damaged nucleosides. Heat-processed foods contain oxidized purines, thymine glycol, and deaminated cytosine or adenine derivatives [9]. Experimental evidence demonstrates that mammalian cells readily uptake such damaged nucleosides, leading to elevated DNA DSBs and chromosomal aberrations in cultured cells and *in vivo* [9]. Hence, salvage provides a direct metabolic route through which dietary DNA damage may intersect with genome integrity.

## Therapeutic implications and opportunities

### Salvage enzyme targets for therapeutic intervention

Several salvage enzymes represent potential therapeutic targets. TK1 is highly upregulated in proliferating cancer cells and is central to the activation of many pyrimidine analog drugs, rendering it a candidate for synthetic lethal strategies in salvage-dependent tumors [140, 141]. dCK activates numerous anticancer nucleoside analogs, including gemcitabine and cytarabine [142]. Inhibition of dCK can sensitize tumors to replication stress [141]. PNP, a key enzyme in purine salvage, has been extensively explored as a therapeutic target in T-cell malignancies [143], and its inhibition may also limit the metabolic activation of damaged purine nucleosides. HPRT1 and APRT, essential for purine base salvage, may be exploited in MYC-driven cancers that exhibit heightened reliance on salvage pathways [144, 145].

### Nucleotide sanitization enzymes as therapeutic modulators

Nucleotide sanitization enzymes offer complementary therapeutic leverage. Inhibition of NUDT1 (MTH1) increases incorporation of oxidized purine nucleotides, driving toxic mispairing in ROS-rich tumors [54], and high level of NUDT15 protein expression is positively correlated with tolerance to 6-mercaptopurine [146]. DUT inhibition elevates dUTP levels, promoting lethal cycles of uracil incorporation and BER [147, 148]. ITPA minimizes incorporation of deaminated purines [43] and 6-thioguanine [149], while SAMHD1 constitutes a biomarker for cytarabine response and a therapeutic target in acute myeloid leukemia [50, 51]. Modulating these enzymes can amplify or suppress salvage-associated genotoxic stress.

### Nucleoside analog therapies

Current cancer therapies already exploit salvage-dependent activation mechanisms. Examples include 5-fluorouracil, 6-thioguanine, azacytidine/decitabine, and gemcitabine, which rely on salvage enzymes for metabolic activation and incorporation into DNA [150–153]. Understanding salvage-driven mutagenesis provides a framework for interpreting differential sensitivity and resistance to these agents.

### Patient-selection biomarkers and precision oncology

Dependence on salvage pathways varies widely across tumors, creating opportunities for biomarker-guided therapy. Candidate biomarkers include expression levels of TK1, dCK, NUDT1, DUT, and SAMHD1 [50, 51, 54, 141, 147], dNTP pool measurements by LC–MS [75], isotope-tracing of salvage flux [141, 154], and mutation spectra indicative of salvage-driven incorporation, such as elevated G→T transversions due to 8-oxodG:dA mispairing [155]. These metrics may guide patient stratification for nucleoside analog therapies or salvage-pathway inhibitors.

### Combination strategies

Aside from targeting individual enzymes or steps within salvage pathways, combinatorial strategies may be particularly effective. Coupling enhanced salvage flux with inhibition of nucleotide sanitization is expected to amplify genotoxic stress. For example, driving incorporation of oxidized nucleosides while blocking NUDT1 [54], or coupling nucleoside analog therapy with checkpoint inhibition to exploit replication stress [156, 157] may constitute viable strategies. Tumors lacking *de novo* synthesis capacity, such as methylthioadenosine phosphorylase (MTAP)-deficient cancers [158], may be particularly vulnerable to such approaches.

## Perspectives and outlook

Nucleotide salvage is essential for sustaining DNA replication and repair when *de novo* synthesis is limited; however, its biochemical permissiveness introduces an overlooked vulnerability for genome stability. By phosphorylating preformed nucleosides, such as oxidized, alkylated, RNA-derived, dietary, and microbiome-associated species, salvage delivers chemically diverse substrates directly to DNA polymerases [6–13]. The broad substrate tolerance of salvage kinases enables metabolic activation of noncanonical nucleosides arising from endogenous metabolism, inflammation, environmental exposure, and diet [1, 2]. Whether these activated substrates accumulate in the dNTP pool is governed by the capacity of nucleotide sanitization enzymes, including NUDT1, ITPA, DUT, and SAMHD1 [41–45, 48], while the balance between replicative and TLS polymerase engagement determines the fidelity of DNA synthesis. Downstream processing by DNA repair pathways, including BER, MMR, NER, and RNase H2-dependent mechanisms, further shapes mutagenic outcomes, replication stress, and genome instability.

Thus far, only a limited subset of modified nucleosides have been assessed for their abilities to undergo metabolic salvage and subsequent incorporation into DNA. Given the diverse array of DNA lesions and RNA modifications, it is important to determine the scope of salvageable substrates. Further studies are needed to uncover which modified nucleosides are efficiently recycled, how they are phosphorylated, and how their incorporation into genomic DNA contributes to mutagenesis, replication stress, and aberrant epigenetic signaling. In this context, isotope tracing and mass spectrometry approaches may provide critical insights into the metabolic fate of modified nucleosides.

Incorporation of modified dNTPs into genomic DNA entails TLS polymerases. These specialized enzymes also allow DNA replication machinery to bypass noncanonical nucleosides, alleviating replication stress often at the expense of accuracy [159, 160]. Thus, the functions of these polymerases are likely vital not only to the incorporation of salvage-derived nucleotides into the genome, but also to the biological consequences of such incorporation. Evaluating the interplay between salvage metabolism and TLS polymerase function will be essential to understand the full consequence of salvage-induced genome instability and may reveal therapeutic opportunities in contexts with heightened dependency on TLS and/or elevated vulnerability to nucleotide salvage, such as cancer [54, 120, 121].

Salvage pathways may also contribute to mutational signatures of unknown etiology, especially in gastrointestinal (GI) cancers. Large-scale sequencing studies have identified recurring single-base substitution (SBS) signatures in GI tract cancers [161–164]. However, the origins of some mutational signatures, such as SBS17a/b, remain unknown [165]. Although some mutational signatures are attributed to oxidative stress and/or exogenous exposure [161–164], salvage-mediated entry of oxidized or alkylated nucleosides into the genome offers an additional plausible route [166]. The salvage of oxidized purines or deaminated pyrimidines may elicit replication errors in specific sequence contexts, potentially aligning with reported, yet unexplained mutational patterns in GI cancers. Moreover, environmental–microbial axis may be especially relevant in GI cancers, where microbiome-derived damaged nucleosides could contribute to such signatures [167]. Hence, a systematic assessment of incorporation of salvage-derived nucleotides into the genome, especially the development of sequencing methods for mapping their incorporation and repair at the genome-wide scale [168–172], may help illustrate new origins of mutational signatures in human cancer as well as other diseases.

Viewing genome instability through the dual lens of direct DNA damage and metabolic recycling opens novel avenues for disease diagnosis, prevention and therapeutic interventions. The salvage pathway, in particular, may represent an important, yet overlooked driver of mutagenesis. Its characterization and management are therefore essential for safeguarding genomic integrity across a spectrum of health and disease states.

## Data Availability

No new data were generated or analyzed in support of this research.
